# Medically Assisted Treatment of Opioid Use Disorder Among Elderly and Non-Elderly Medicare Beneficiaries

**DOI:** 10.1093/geroni/igaa057.175

**Published:** 2020-12-16

**Authors:** Jennifer Miles, Stephen Crystal, Peter Treitler, Richard Hermida

**Affiliations:** Rutgers University, New Brunswick, New Jersey, United States

## Abstract

Although medication for addiction treatment (MAT) is known to be the most effective treatment for opioid use disorder (OUD), these medications are widely underutilized, especially among older adults and racial/ethnic minorities. Of the three main MAT modalities, Medicare covered buprenorphine and naltrexone in 2017; methadone was not covered until 2020. We examined MAT prescribing among elderly compared with non-elderly Medicare beneficiaries. Our sample was drawn from a ~40% random sample of 2017 Medicare beneficiaries with Part D coverage and was comprised of elderly beneficiaries (age 65+) with OUD (N=112,314) or who experienced opioid poisoning (N=9,657), and non-elderly Medicare beneficiaries (the Medicare disability population, age 0-64) with OUD (N=161,423) or opioid poisoning (N=13,591). MAT was underutilized in both Medicare populations, but especially in the elderly population. Of elderly beneficiaries with OUD, 5.1% and 0.8% were prescribed buprenorphine and naltrexone, respectively, compared to 15.5% and 2.3% among non-elderly. Among elderly beneficiaries with opioid poisoning, 3.1% and 0.8% were prescribed buprenorphine and naltrexone, respectively, compared to 10.1% and 3.2% in the non-elderly population. Sharp racial/ethnic disparities were identified within each age group. These findings highlight the need to expand access to MAT for Medicare beneficiaries, particularly older adults among whom underutilization is pronounced. Several recent Medicare policy changes have sought to address this issue, but continuing efforts and close monitoring are warranted in an effort to dramatically increase rates of treatment for elderly with opioid use disorder.

